# A longitudinal pilot study examining the influence of the orthodontic system chosen in adult patients (brackets versus aligners) on oral health-related quality of life and anxiety

**DOI:** 10.1186/s12903-024-04464-7

**Published:** 2024-06-27

**Authors:** Laura Correa, Alberto Albaladejo, Adrián Curto

**Affiliations:** Faculty of Medicine, University of Salamanca, Salamanca, Spain

**Keywords:** Orthodontics, Brackets, Aligners, Oral health-related quality of life, Anxiety

## Abstract

**Background:**

In recent years, the demand for orthodontic treatment with aligners has increased, led by patient need, as aligners typically provide them with improved aesthetics and less physical discomfort. In deciding with the patient on an appropriate orthodontic system, it is important to take into account the potential discomfort and the perceptions that patients have in relation to their treatment. The objective of this study was to analyze the influence of brackets or aligners on oral health-related quality of life (OHRQoL) and anxiety levels in a sample of adult patients during the first month of treatment.

**Methods:**

The pilot study was carried out at the Dental Clinic of the University of Salamanca between November 2023 and February 2024. Eighty adult patients who initiated orthodontic treatment were selected and divided into two groups: the brackets group (Victory®; 3 M Unitek, California, USA) (*n* = 40) and the aligners group (Invisalign®; Align Technology, California, USA) (*n* = 40). OHRQoL was analyzed using the Oral Health Impact Profile-14 (OHIP-14) questionnaire, and anxiety was analyzed using the State–Trait Anxiety Inventory (STAI). The follow-up time was one month, with scores recorded at the beginning (T0) and one month after starting treatment (T1).

**Results:**

The mean patient age was 33.70 (± 5.45) years old. The total sample (*n* = 80) consisted of 66.2% men and 33.8% women. In the brackets group, one month after starting treatment, the dimension with the highest impact was that of physical pain (5.62 ± 1.51). In the aligners group, where the dimension of psychological disability had the highest score (4.22 ± 1.02). In the brackets group the total OHIP score was higher at one month (T1) (33.98 ± 6.81) than at the start of treatment (T0) (21.80 ± 3.34); this greater impact on OHRQoL one month after starting treatment was not observed in the aligners group (T1 = 27.33 ± 6.83; T0 = 27.33 ± 6.22). The orthodontic system used did not influence participants’ anxiety (*p* > 0.05). Age and sex were not influential factors in either OHRQoL or anxiety.

**Conclusions:**

The bracket system significantly influenced patients’ OHRQoL. In the sample studied, no influence of the orthodontic system (brackets versus aligners) on anxiety was observed.

## Background

The demand for orthodontic treatments has increased because of the possibility of using aligners in treatment, principally due to patients’ aesthetic demands [1]. This improvement in aesthetics has produced a concomitant increase in patients’ oral health-related quality of life (OHRQoL) [2, 3].

Aligners were developed as an aesthetic orthodontic alternative to the use of fixed brackets [1, 4]. Aligners also facilitate significantly improved oral hygiene, less discomfort, and greater convenience for patients, compared to fixed brackets; therefore, they can reduce the adverse effects of orthodontic treatment compared to conventional fixed brackets [5, 6].

In recent years, there has been an increased interest in research on OHRQoL and the anxiety that patients may experience during their orthodontic treatment [7]. The presence of a malocclusion has been observed as negatively affecting OHRQoL, especially at the beginning of orthodontic treatment. Patients typically request orthodontic treatment to improve their dental aesthetics, oral functionality, and psychosocial well-being [8, 9].

The scientific literature has concluded that orthodontic treatment can improve or worsen patients’ OHRQoL depending on which phase of treatment the patient is in. The impact of orthodontic treatment on OHRQoL decreases as treatment progresses. Evaluating OHRQoL may be an effective means of analyzing the results of orthodontic treatment in patients [8, 10, 11].

The pain that patients describe during orthodontic treatment can be influenced by different factors, including psychological traits such as their innate response to stress [12–14]. Different studies have reported that the pain described by patients also varies depending on the orthodontic system used. Treatment with brackets usually produces more pain compared to treatment with aligners [15, 16]. The discomfort and pain described by patients during the early stages of orthodontic treatment, especially during the first month, have a negative influence on patients’ OHRQoL and on their anxiety when facing treatment. This impact can have a negative repercussion on patients’ compliance with indications and may even make them unwilling to continue orthodontic treatment [10, 11, 13].

There is limited scientific evidence that analyzes the impact of orthodontic treatment on OHRQoL and the anxiety that patients describe during the early stages of treatment. The impact of orthodontic treatment on OHRQoL and anxiety levels during the first month of orthodontic treatment needs to be analysed. This first month of orthodontic treatment has a negative influence on patients’ pain levels [2, 7, 8].

Therefore, the objective of this pilot study was to analyze the possible influence of the orthodontic system used (brackets compared to aligners) on OHRQoL and anxiety levels in adult patients during the first month of treatment. The experimental hypothesis of this study is that OHRQoL and anxiety levels differ between patients treated with brackets and aligners during the initial phase of orthodontic treatment.

## Methods

### Study design

This pilot study was approved by the Research Ethics Committee of the University of Salamanca (study reference number: 1074). This study followed the ethical principles established by the Declaration of Helsinki for research with humans, and the STROBE guidelines for conducting observational studies [17].

The patients were informed of the examination procedures and were guaranteed confidentiality of their collected information. Before recruitment, signed consent was obtained from each participant.

### Interventions

OHRQoL and anxiety were analyzed in a consecutive sample of 80 patients who began orthodontic treatment at the Dental Clinic of the University of Salamanca. This sample was divided into two study groups: the brackets group (*n* = 40) and the aligners group (*n* = 40). No patient dropped out of the study while it was ongoing.

The participants in the brackets group were bonded with 0.022-inch slots MBT prescription stainless steel brackets (Victory^®^; 3 M Unitek, California, USA) in both arches. In the first clinical appointment, the upper and lower brackets and the tubes of the first permanent molars were cemented. The archwire was 0.014-inch NiTi (Ormco, California, USA) at baseline. The type of engagement with the elastomeric ligature (Dentaurum GmbH & Co., KG, Ispringen, Germany) was identical for all of the patients.

In the group of patients with aligners, the Invisalign^®^ system (Align Technology, California, USA) was used. Tooth movements were planned at the rate recommended using algorithms from the ClinCheck Pro program. In the first clinical appointment, the attachments were cemented and the aligners were delivered. The patients were instructed to change aligners every seven days.

### Eligibility criteria for participants

The inclusion criteria were as follows: adult patients (> 18 years); patients with permanent dentition (except third molars); and a maximum Little’s irregularity index of 6 mm.

The exclusion criteria were as follows: patient history of previous orthodontic treatment; orthodontic treatment with extractions; patients with craniofacial anomalies; patients with untreated caries; patients with untreated gingival and/or periodontal pathology; patients with symptoms of or diagnosed temporomandibular joint pathology; patients receiving treatment with anti-inflammatory drugs, analgesics, anxiolytics, and/or antidepressants; and pregnant patients.

### Outcome measures

The impact of orthodontic treatment on patients’ OHRQL was analyzed using the Spanish version of the Oral Health Impact Profile-14 (OHIP-14) questionnaire [18].

The OHIP-14 questionnaire consists of 14 items that analyze the following seven domains of OHRQL: functional limitation, physical pain, psychological discomfort, physical disability, psychological disability, social disability, and disability. The responses to this questionnaire were scored using a 5-point Likert scale (0 = never, 1 = almost never, 2 = occasionally, 3 = quite often, and 4 = very often) [19].

Anxiety was assessed with the State–Trait Anxiety Inventory (STAI). The STAI is a self-reported inventory that encompasses two independent scales that measure state anxiety (STAI-S) (how one feels at a given time) and trait anxiety (STAI-T) (how one usually feels) [20]. The Inventory is a 40-item Likert scale that evaluates separate dimensions of anxiety as a state (items 1–20) and anxiety as a trait (items 21–40). A score greater than 40 points is an indicator of a high degree of anxiety [20, 21].

The OHIP-14 and STAI questionnaires were provided to all study participants and completed at baseline (T0) and one month after starting treatment (T1).

### Statistical analysis

Data were analyzed with the SPSS version 28 program (SPSS Inc., Chicago, IL, USA). Qualitative variables were analyzed with tables of frequencies, percentages, Student’s *t*-test, and the Chi-square test. We selected a significance level of *p* < 0.05.

## Results

### Baseline data

The sample analyzed consisted of 80 patients divided into two study groups: 40 participants (30 men, 10 women) with a mean age of 32.15 years (± 5.79) in the brackets group, and 40 participants (23 men, 17 women) with a mean age of 35.25 years (± 4.67) in the aligners group. The descriptive statistics of the characteristics of the participants are shown in Table 1.

**Table 1:** Patient baseline characteristics

	Total (*n* = 80)	Brackets Group (*n* = 40)	Aligners Group (*n* = 40)	*p*-value
Age (years), mean (SD)	33.70 (± 5.45)	32.15 (± 5.79)	35.25 (± 4.67)	Student’s *t* = 0.010^*^
Sex, n (%)				
Male	53 (66.2%)	30 (75.0%)	23 (57.5%)	Chi^2^ = 0.098^NS^
Female	27 (33.8%)	10 (25.0%)	17 (42.5%)	

NS = Not significant (*p* > 0.05); * = Significant (*p* < 0.05)

### Oral health-related quality of life analysis

We analyzed the significance of the changes in the OHRQoL variables between the first month of treatment (T1) and at the start (T0) in the study population (*n* = 80). In the total sample, an increase in the negative impact of orthodontic treatment on OHRQoL was observed one month after starting treatment (T1), compared to the beginning (T0). The differences between T1 and T0 in the variables of the OHIP-14 questionnaire were all statistically significant (*p* < 0.01). The dimension of physical pain (+ 1.38) showed the most significant variation compared to the dimension of psychological discomfort, which showed the least variation (+ 0.56) (Table 2).

**Table 2:** Comparison of OHRQoL between baseline and the follow-up period, for the total sample

OHIP-14	Mean (SD)		Student’s t-test	*p*-value
	T0 (*n* = 80)	T1 (*n* = 80)		
Functional limitation	3.70 (± 1.13)	4.61 (± 1.51)	4.66	< 0.01^**^
Physical pain	3.48 (± 1.06)	4.85 (± 1.71)	6.62	< 0.01^**^
Psychological discomfort	3.65 (± 1.08)	4.21 (± 1.35)	3.04	< 0.01^**^
Physical disability	3.48 (± 0.99)	4.28 (± 1.21)	5.40	< 0.01^**^
Psychological disability	3.58 (± 1.18)	4.58 (± 1.18)	5.43	< 0.01^**^
Social disability	3.48 (± 1.12)	4.21 (± 1.28)	3.84	< 0.01^**^
Disability	3.21 (± 1.41)	3.91 (± 1.03)	3.68	< 0.01^**^
Total OHIP	24.56 (± 5.69)	30.65 (± 7.56)	6.15	< 0.01^**^

** = Highly significant (*p* < 0.01)

**Fig. 1:**
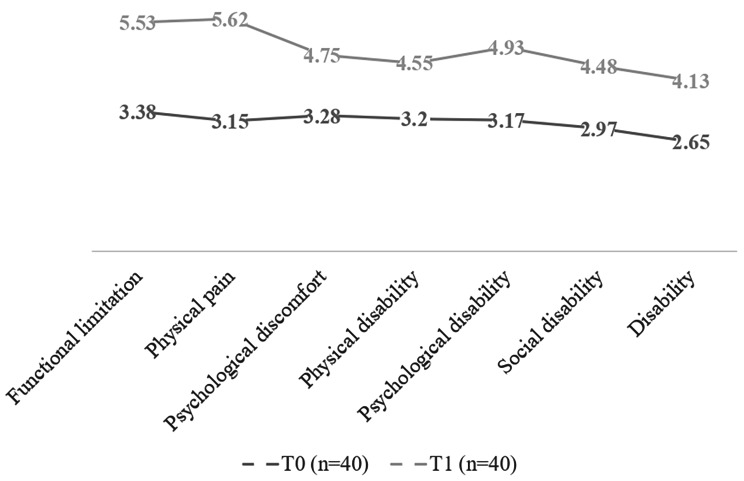
Evolution of OHRQoL in the brackets group during the first month of treatment

In the brackets group, statistically significant differences (*p* < 0.01) were observed in all dimensions and in the total OHIP-14 score, when comparing the scores after a month and at the beginning of treatment. In this group, in the first month of treatment, the brackets had a negative impact on patients’ OHRQoL. The dimension that had the most significant impact one month after starting treatment was that of physical pain, compared to the disability dimension that had the least impact (Table 3) (Fig. 1).

**Table 3:** Comparison of OHRQoL between baseline and the follow-up period, in the brackets group

OHIP-14	Mean (SD)		Student’s t-test	*p*-value
	T0 (*n* = 40)	T1 (*n* = 40)		
Functional limitation	3.38 (± 1.03)	5.53 (± 1.41)	10.07	< 0.01^**^
Physical pain	3.15 (± 0.92)	5.62 (± 1.51)	9.59	< 0.01^**^
Psychological discomfort	3.28 (± 0.93)	4.75 (± 1.26)	6.28	< 0.01^**^
Physical disability	3.20 (± 0.82)	4.55 (± 1.15)	6.82	< 0.01^**^
Psychological disability	3.17 (± 0.98)	4.93 (± 1.23)	7.39	< 0.01^**^
Social disability	2.97 (± 1.19)	4.48 (± 1.13)	5.65	< 0.01^**^
Disability	2.65 (± 1.10)	4.13 (± 1.02)	6.77	< 0.01^**^
Total OHIP	21.80 (± 3.34)	33.98 (± 6.81)	12.17	< 0.01^**^

** = Highly significant (*p* < 0.01)

**Fig. 2:**
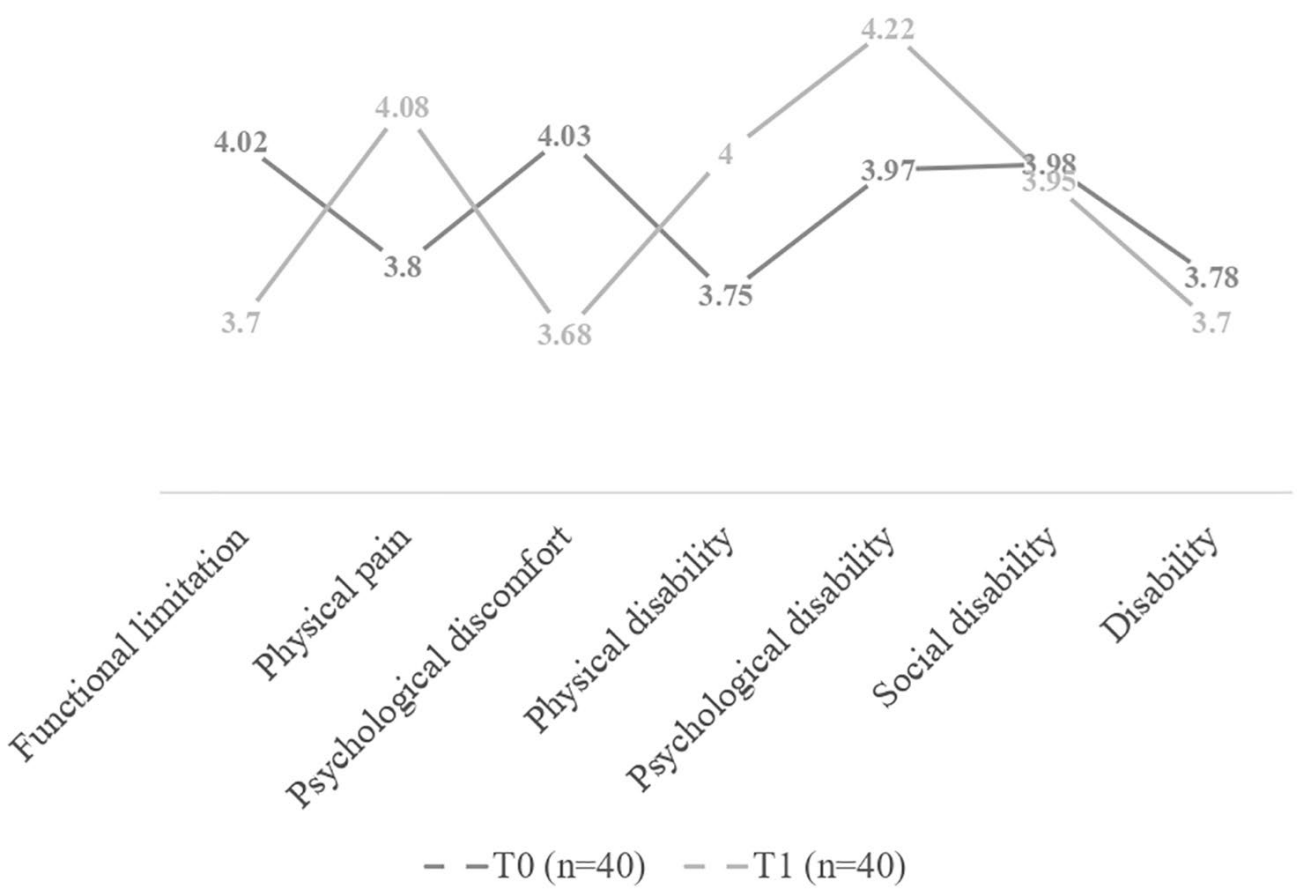
Evolution of OHRQoL in the aligners group during the first month of treatment

In the aligners group, compared to the patients with brackets, no significant differences were observed in any of the dimensions or in the total OHIP-14 score one month after starting treatment. A slight worsening in OHRQoL was observed in these patients, but this was not clinically significant. One month after starting orthodontic treatment, the dimension with the most significant impact was psychological disability, compared to the dimensions of functional limitation and disability, which were the items with the least impact on OHRQoL (Table 4) (Fig. 2).

**Table 4:** Comparison of OHRQoL between baseline and the follow-up period, in the aligners group

OHIP-14	Mean (SD)		Student’s t-test	*p*-value
	T0 (*n* = 40)	T1 (*n* = 40)		
Functional limitation	4.02 (± 1.14)	3.70 (± 0.94)	1.84	0.074^NS^
Physical pain	3.80 (± 1.09)	4.08 (± 1.54)	1.28	0.208^NS^
Psychological discomfort	4.03 (± 1.10)	3.68 (± 1.23)	1.74	0.090^NS^
Physical disability	3.75 (± 1.08)	4.00 (± 1.22)	1.35	0.185^NS^
Psychological disability	3.97 (± 1.23)	4.22 (± 1.02)	1.09	0.281^NS^
Social disability	3.98 (± 0.80)	3.95 (± 1.38)	0.11	0.911^NS^
Disability	3.78 (± 1.48)	3.70 (± 1.02)	0.29	0.776^NS^
Total OHIP	27.33 (± 6.22)	27.33 (± 6.83)	0.00	1.0^NS^

NS = Not significant (*p* > 0.05)

Sex and age did not have a statistically significant influence on the impact of orthodontic treatment on OHRQoL in the sample analyzed.

### Anxiety analysis

No significant influence of orthodontic treatment on anxiety levels was observed one month after starting treatment (T1). A slight decrease in anxiety was observed one month after starting treatment, but this was not clinically significant (Table 5).

**Table 5:** Comparison of anxiety between baseline and the follow-up period, for the total sample

STAI	Mean (SD)		Student’s t-test	*p*-value
	T0 (*n* = 80)	T1 (*n* = 80)		
Anxiety-State	30.66 (± 2.38)	30.05 (± 2.52)	1.44	0.155^NS^
Anxiety-Trait	29.15 (± 2.79)	28.45 (± 2.98)	1.95	0.052^NS^

NS = Not significant (*p* > 0.05)

The scores on the STAI inventory were compared between the two study groups. At the beginning of treatment, in the anxiety-trait, a significant difference was observed between the two study groups; however, clinically, this small difference was not important (Table 6).

**Table 6:** Comparison of the changes in the STAI questionnaire’s variables between treatment groups

STAI	Mean (SD)		Student’s t-test	*p*-value
	Brackets group (*n* = 40)	Aligners group (*n* = 40)		
Anxiety-State (T0)	30.65 (± 1.79)	30.67 (± 2.87)	0.04	0.963^NS^
Anxiety-Trait (T0)	28.32 (± 2.70)	29.98 (± 2.66)	2.75	0.007^**^
Anxiety-State (T1)	29.90 (± 2.54)	30.20 (± 2.51)	0.53	0.597^NS^
Anxiety-Trait (T1)	27.83 (± 2.92)	29.08 (± 2.94)	1.90	0.060^NS^

NS = Not significant (*p* > 0.05); ** = Highly significant (*p* < 0.01)

There were no influences of patients’ sex and age on their levels of anxiety in this study.

## Discussion

The scientific literature includes only a few publications on the anxiety and OHRQoL of patients treated with aligners [11, 22]. Therefore, this longitudinal pilot study aimed to analyze the impact of orthodontic treatment with aligners on OHRQoL and anxiety compared to the bracket system, during the first month of orthodontic treatment.

There were no statistically significant differences between the participants of the brackets group and the aligners group in terms of their sex; additionally, whilst significant differences in age were observed between the groups, they were not clinically important. Therefore, the two study groups were considered homogeneous in terms of their sociodemographic characteristics and the severity of their malocclusion.

In this study, the OHIP-14 questionnaire was used to analyze the OHRQoL. This instrument is considered a valid tool for evaluating OHRQoL in patients undergoing orthodontic treatment [22–26].

To analyze the impact of orthodontic treatment with brackets or aligners on anxiety, we used the STAI inventory. This tool has also been used in previous studies in orthodontics [12, 13, 27, 28].

Orthodontic treatment can significantly improve patients’ OHRQoL [19, 23, 24, 29]. In this study, in the total sample, it was observed that at one month after starting treatment, the patients’ OHRQoL was worse than at the beginning. However, in the aligners group, in the dimensions of functional limitation, psychological discomfort, social disability, and disability measured with the OHIP-14 questionnaire, a trend appeared in which one month after starting treatment with aligners, the patients showed a lower degree of impact on all of these dimensions compared to the start of treatment, but without statistically significant differences. One month after starting treatment, the brackets group described a higher total score on the OHIP-14 questionnaire (33.98 ± 6.81) than the aligners group (27.33 ± 6.83). These results can be related to the fact that, during their treatment with aligners, patients were able to remove the aligners during meals. In the group of patients with brackets, one month after starting treatment (T1), the dimension of the OHIP-14 questionnaire with the highest score was that of physical pain (5.62 ± 1.51). This fact can be explained due to the discomfort that brackets can cause in patients (for example, the appearance of oral wounds). These results coincide with those reported by other authors [22, 29, 30].

Previous studies have also analyzed the impact of orthodontic treatment on OHRQoL, comparing patients being treated with brackets and patients being treated with aligners. These studies observed, as in this case, that treatment with aligners produced a lower impact on OHRQoL of adult patients compared to the bracket system, one month after starting treatment (brackets = 33.98 ± 6.81; aligners = 27.33 ± 6.83). Alfawal et al. [24] recorded that patients with aligners described a lower total score measured with the OHIP-14 questionnaire (14.14 ± 3.66) compared to patients with brackets (25.18 ± 4.15) one month after starting treatment. Similarly, results were obtained in the study of Jaber et al. [31], where one month after starting treatment, the group of patients with aligners described a lower total score on the OHIP-14 (5.82 ± 3.96) compared to patients with brackets (14.12 ± 9.07).

There are very few previous studies that have evaluated the possible influence of the orthodontic system used (brackets versus aligners) on anxiety levels. Gao M et al. [22] evaluated the influence of the orthodontic system (brackets compared to aligners) on the anxiety-state levels of the STAI inventory. They concluded that therapy with aligners produced lower levels of anxiety in patients compared to the use of brackets (*p* < 0.05) from the start of treatment to the end of the 14th day [22]. In our study, anxiety-trait levels with significant differences were only observed at baseline (T0). One month after starting treatment (T1), no statistically significant differences were observed between the two treatment systems.

In the present study, it was observed that anxiety levels were lower one month after starting treatment compared to the beginning, in both study groups. These results are in agreement with those reported by other authors, such as Wang et al. [13], who analyzed anxiety levels in patients who started orthodontic treatment with brackets and also concluded that anxiety levels were lower at one month (STAI = 31.0) compared to the start of treatment (STAI = 38.0); however, these authors did not observe a substantial decrease in anxiety levels between the first month and the start, as in our study.

Sex has been reported to be an influential factor on anxiety in the general population [32]. In this study, we observed that sex and age did not influence OHRQoL or anxiety levels. These results are similar to those reported in previous studies [33–35].

The results described in this study can provide information for orthodontists to provide to patients before starting their orthodontic treatment. Providing this information can increase patient cooperation and understanding during their orthodontic treatment. Knowing in advance the discomfort of orthodontic treatment can help professionals psychologically prepare the patient before starting their treatment.

### Limitations

One of the limitations of this study is that the follow-up period was only one month, since the objective of this work was to analyze only the initial phases of orthodontic treatment. Another limitation of this study was that the type of malocclusion of the patients participating in the study was not taken into account. The degree of severity of malocclusion may influence patients’ OHRQoL and anxiety levels.

Randomized clinical studies are necessary, with a follow-up period appropriate to the duration of orthodontic treatment, to validate the effects of treatment with brackets and aligners on OHRQoL and anxiety levels. Analysing participants from different demographic groups can provide interesting practical information. It would be interesting to carry out multicenter research to analyze the different factors that may influence anxiety and OHRQoL, as well as to analyze the effects of the use of analgesic drugs during treatment on OHRQoL and anxiety.

## Conclusions

- Bracket treatment had a negative influence on patients’ OHRQoL one month after starting treatment.
- The use of aligners did not influence OHRQoL one month after starting treatment.
- The orthodontic system used did not influence anxiety levels during the first month of treatment.
- In the sample analyzed, neither sex nor age influenced OHRQoL or anxiety described by patients.

## Data Availability

The datasets used and/or analysed during the current study available from the corresponding author on reasonable request.

## Abbreviations

NS: Not significant
OHRQoL: Oral-health-related quality of life
SD: Standard deviation
STAI: State trait anxiety inventory
STROBE: Strengthening the reporting of observational studies in epidemiology
